# Died

**Published:** 1856-05

**Authors:** 


### DIED:

Of a long speech, so some one writes of General Taylor, that a long speech of some self appreciating orator effected what the fierce artillery of Buena Vista failed to accomplish. Many facts are interesting, beautiful, or striking until they are falsified. And so with the statement which we have here recorded.

The long speech was a part, a small part of the causes which left General Taylor in a state of bodily exhaustion at the close of the ceremonies of laying the corner stone of a public building, the Washington Monument, on a hot day in midsummer. If the patriot warrior had gone immediately home and had taken a cup of strong red pepper tea, or other similar warm drink, and had lain down with a slight cover over him for a "nap" of nature's own apportioning, it is scarcely possible that he should not have waked up next morning in his usual health. But he did not do this, he ate a bowl of fruit or berries with milk, which was either cold itself, or was accompanied with iced drinks, and in a short time thereafter, against the remonstrances of a physician in the company, he ate a hearty dinner resulting in cholera and death.

The cold drinks taken into the stomach as certainly chilled the body and closed the pores on the surface as if they had been dashed over him; nature in her alarm turned the drain in upon the bowels, but the exhaustion of the day's exercise had taken away the power of reaction, and all was lost. More probably there would have been a reaction, had not the nervous energy remaining been compelled away to aid the laboring stomach to dispose of the unfortunate dinner.

A large amount of suffering, and sickness, and death, result every year from eating or drinking heartily under circumstances of great bodily exhaustion, or prostration, or mental anxiety or distress. We give it as a piece of advice, worth a life, eat nothing under circumstances of high mental excitement, or great bodily weakness, drink nothing, (not even the renowned "glass of brandy and water") except a cup or two or more of strong red pepper tea, as warm as practicable, and wait until a little rest and sleep, nature's blessed remedies, have somewhat restored you.

